# The 510(k) Third Party Review Program: Promise and Potential

**DOI:** 10.1007/s10916-023-01986-5

**Published:** 2023-08-29

**Authors:** Brian J. Miller, William Blanks, Brian Yagi

**Affiliations:** 1 Division of Hospital Medicine, Department of Medicine, The Johns Hopkins University School of Medicine, 600 N. Wolfe Street, Meyer 8-143, Baltimore, MD 21287 USA; 2 The Johns Hopkins Carey Business School, Baltimore, MD USA; 3 American Enterprise Institute, Washington, DC USA; 4 West Virginia University School of Medicine, Morgantown, WV USA

**Keywords:** Medical device regulation, FDA, 510(k), Health policy

## Abstract

Every year, the Food and Drug Administration (FDA) clears approximately 3,000 medical devices for marketing via the 510(k) pathway. These constitute 99% of all devices approved for human use and includes the premarket review of many devices incorporating newer technology such as artificial intelligence (AI), machine learning (ML), and other software. As the complexity of these novel technologies and the number of applications is expected to increase in the coming years, statutory changes such as the 2016 21^st^ Century Cures Act, regulations, and guidance documents have increased both the volume and complexity of device review. Thus, the ability to streamline the review of less complex, low-to-moderate risk devices through the 510(k) pathway will maximize the FDA’s capability to address other important, future-oriented regulatory questions. For over twenty five years, third party review organizations have served a defined function to assist with the review of 510(k) applications for a set of enumerated device classes. This paper reviews the history of FDA device regulation, the evolution of the 510(k) review pathway, and the recent history of the 510(k) third party review program. Finally, the paper addresses policy concerns from all stakeholders – including the FDA – along with policy suggestions to improve the third party review program and FDA device regulation writ large.

Every day in homes and health care facilities across the country patients and physicians utilize or depend upon a product from one of over 6,700 U.S. Food and Drug Administration (FDA)-regulated medical device products. The economic scale is also significant: the medical device market is estimated to account for over $176 billion (2020) in domestic annual sales [1]. Medical devices are subject to FDA regulatory authority and premarket review under the 1976 Medical Device Amendments to the Federal Food, Drug, and Cosmetic Act (FD&C Act) [2], with device regulation activities comprising one-tenth of the FDA’s annual budget [3]. The FDA determines the evidentiary burden required for marketing approval as well as the distribution of pre- and post-market regulatory risk through a variety of regulatory pathways.

This review article briefly covers the basics of the FDA market entry pathway for medical devices (a topic explored in more detail in other reviews) [4], with a focus on the use and function of the 510(k) pathway. After reviewing the 510(k) pathway, the article reviews the creation and history behind the 510(k) third party review program (formally called the “Accredited Persons Program,” as the third party review organizations need to be formally accredited by the FDA) [5] along with its performance to date. Finally, the article reviews potential policy improvements to the third party review program designed to improve the efficiency of FDA medical device regulation, thus freeing up internal agency resources for complex product reviews and over-the-horizon regulatory efforts targeting emerging complex product categories.

## FDA medical device regulation and the 510(k) review pathway

The FDA’s Center for Devices and Radiological Health (CDRH) oversees devices that are meant to diagnose, cure, mitigate, treat, or prevent disease [6]. A device could be an implant, component, accessory, or other instrument and includes everything from stethoscopes to electrosurgical equipment to knee implants. In some low-risk product areas such as medical device data systems and medical image storage devices [7], software that automates simple tasks for health care providers, and software that helps patients self-manage their disease or conditions [8], the FDA has chosen to exercise enforcement discretion and does not require pre-market review and authorization. Additionally, the 21^st^ Century Cures Act defined the scope of the FDA’s regulatory authority over software (including, but not limited to artificial intelligence or AI). Specifically, it carved out 5 functions or uses of software that would be exempted from review as a medical device: administrative support, healthy lifestyle/wellness, electronic patient records, transfer/storage of medical device data, and certain types of clinical decision support [9].

In all other areas, the FDA oversees medical devices through a risk-based regulatory system created under the 1976 Medical Device Amendments and revised through successive legislative efforts. Class I or low risk devices (e.g. bandages, nonelectric wheelchairs) are subject to so called general controls, such as current good manufacturing practices based upon a quality system, device registration and listing, premarket notification, and other basic regulatory tools [10]. Class II or moderate-risk devices such as magnetic resonance imaging (MRI) scanners or intravenous medication infusion pumps necessitate pre-market notification and require special controls [11] such as meeting FDA-recognized performance standards or certain post-market surveillance requirements. Class III or high-risk devices such as pacemakers require a pre-market approval (PMA) application including the submission of clinical trials to provide support for the assurance that the device is safe and effective. Changes or updates to a class III device occur via submission of a supplement, or sPMA.

**Fig. 1:**
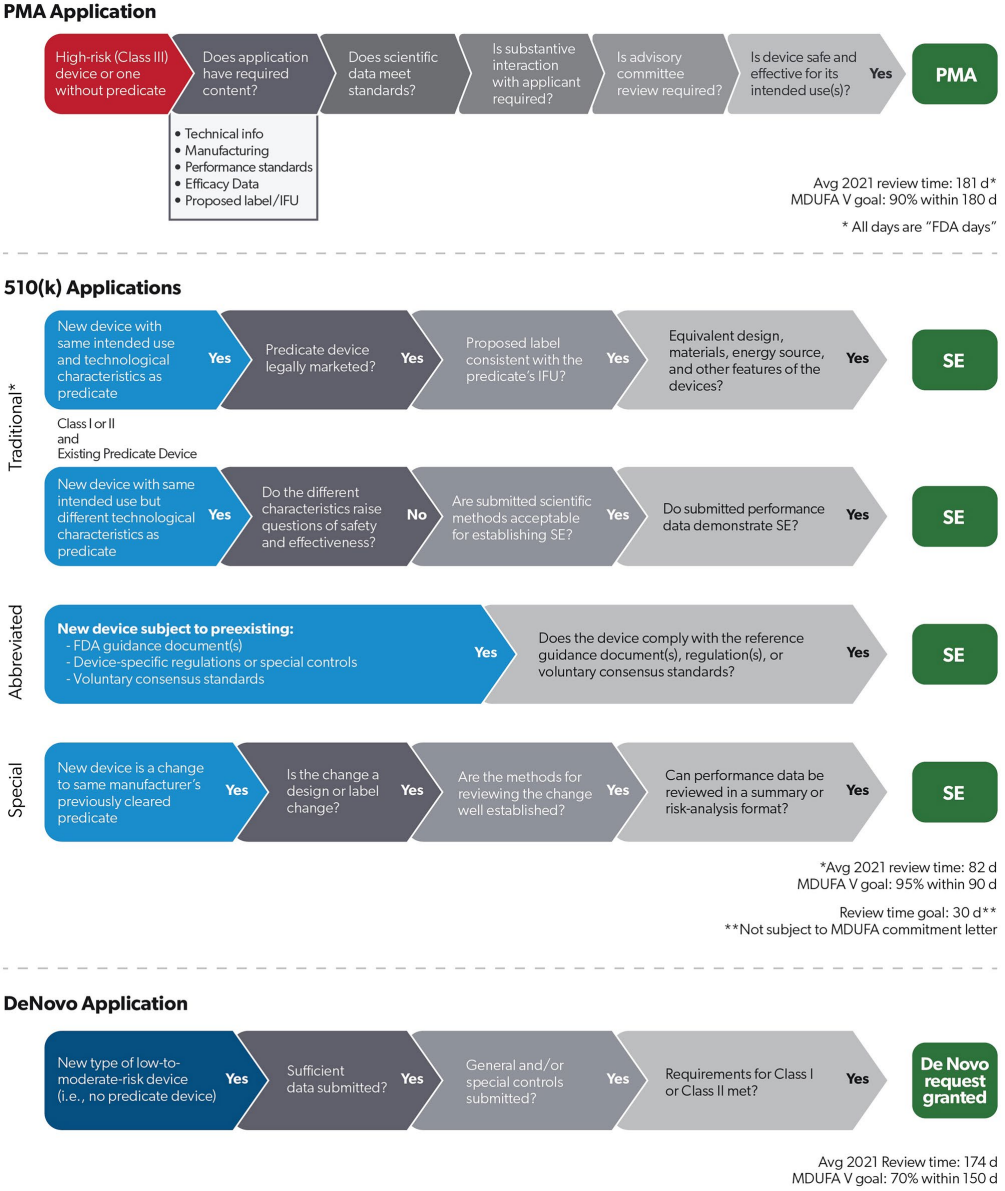
Premarket device review pathways. Prior to legally marketing a medical device, the manufacturer must apply for authorization through one of the statutorily enacted pathways. The PMA pathway is for the highest risk devices (Class III). A new device with a similar characteristics and intended use as a device that was previously approved (i.e., a predicate device) can apply through the 510(k) pathway. If there is no predicate device, the new device is technically classified as a Class III device and must either be approved through the PMA pathway or through the De Novo pathway. The FDA has released Guidance documents to advise industry on the elements required in each application as well as the requirements for the agency’s substantive review. The PMA pathway is the most stringent, with the highest evidentiary burden (usually requiring clinical safety and efficacy data) and takes the FDA, on average, 181 days to reach a decision. De Novo applications take an average of 174 days to review, while traditional 510(k) applications take an average of 82 days to review [21]. The FDA sets review time goals with each quinquennial reauthorization of the Medical Device User Fee Act, most recently iteration V in 2022 [38]. * All days are reported as “FDA days” (i.e., all calendar days, including weekends and holidays, after an application has been submitted to the FDA and is under the agency’s review). Key: *PMA* Premarket Approval, *SE* Substantial Equivalence, *MDUFA* Medical Device User Fee Act, *IFU* Indication for Use

Devices seeking marketing authorization after 1976 are by default considered class III and require submission and approval of a PMA. If a manufacturer can demonstrate that a class I or II device is substantially equivalent to a previously marketed device – a predicate device – then the manufacturer can instead file a 510(k) submission (see Fig. 1). A 510(k) submission requires that a company demonstrate that a device has the same intended use and technological characteristics as the predicate device. Alternatively, the manufacturer may demonstrate that the device has the same intended use, but different characteristics that do not raise safety or efficacy questions. In this situation, the FDA will usually request performance data to support the application and will scrutinize the performance study’s scientific methods in addition to its findings on safety and efficacy [12]. Manufacturers are required to notify the agency 90 days in advance of marketing a device.

Device manufacturers can choose a lane best suited to their product from amongst multiple iterations of the 510(k) pathway, each with their own specific use. The De Novo 510(k) pathway, created in 1997, updated legislatively in 2012, and clarified in 2022 rulemaking [13], allows a manufacturer to file a 510(k) for a Class I or II device for which there is no predicate. A De Novo request may be submitted when a manufacturer receives a determination of not substantially equivalent for a previously filed 510(k) application or, alternatively, the manufacturer may file a de novo request without first filing a 510(k) application. The Abbreviated 510(k) program offers manufacturers a related flexibility: manufacturers can demonstrate substantial equivalence to a predicate device by providing the results of testing demonstrating that the device comports with previously recognized FDA consensus standards [14], now numbering nearly 1,500 [15]. Finally, the Special 510(k) pathway [16] allows a manufacturer to receive expedited review within 30 days and rely on the prior review of its submission when making changes to a previously marketed device to be deemed substantially equivalent. Regulatory decisions are based upon review of design control procedures or with performance data using an FDA-designated well-established method (e.g. FDA-recognized consensus standard, well-established scientific method, etc.) and presented in a summary or risk-analysis format [17].

The 510(k) pathway is responsible for ~ 99% of device reviews. A study examining a decade of device reviews (2008 – 2017) noted that the regulatory market share of PMA approvals rose from 0.7 to 1.5%, with an annual mean of 31 devices approved via a PMA compared to 2,825 devices cleared via the 510(k) pathway. The study also found that class I recalls (those with reasonable probability of serious adverse health event or death) represented 5.2% and 0.8% of each regulatory marketplace, respectively, a difference that was statistically significant noting that differences also exist across therapeutic areas [18]. These statistics support the regulatory paradigm that PMAs focus on revolutionary innovation while the 510(k) pathway centers on incremental innovation.

Criticisms of the 510(k) pathway are varied. Industry, with user fees at $19,870 or $4,967 for small businesses [19], notes the challenges of timeliness of FDA meeting statutory goals for review of 510(k) applications, with 89% of (3,268) FY2021 510(k) premarket notifications, 82% of (187) 180-day PMA supplements requiring substantive interaction, and 41% of (49) De Novo decisions meeting their current MDUFA review goals [20]. Inefficiency of regulatory review delays market entry, potentially depriving clinicians and patients of incremental gains in device design, in addition to incurring costs for manufacturers large and small.

Progressive public policy advocates note concerns of predicate scope creep or drift, [22] loosely defined as when a recently 510(k)-cleared device is substantially different in form or use from its original predicate device due to numerous interval rounds of incremental innovation via the 510(k) pathway. Still, other academic researchers denote specific cases of concern [23, 24], arguing there is an association between safety issues and the incremental evidentiary approach underlying the 510(k) clearance process. A 2011 Institute of Medicine (IOM) Report recommended the creation a new integrated premarket and post-market regulatory framework for Class II devices [25], a policy alternative unlikely to be executed due to policy and political barriers, further noting that the IOM committee lacked representation from the entrepreneurial, medical technology, and medical device communities. Finally, despite these concerns a diverse set of stakeholders [26–30] including the FDA itself [31] have declared the need to spend more time, energy, and human capital to address future “around the corner” regulatory issues such as the regulation of artificial intelligence/machine learning (AI/ML), software as a medical device (SaMD), and the blending of software and traditional medical devices.

Woven into the background of the aforementioned policy concerns is a persistent, well-documented agency challenge in hiring qualified technical staff to support statutorily-mandated agency review programs. FDA human capital acquisition and retention, the subject of many critical reports [32, 33], is buttressed by specific hiring authorities and salary flexibility to award relative differential compensation above the standard civil service pay scale to technical experts such as physicians and engineers. Barriers include the high cost of living in the Washington-Baltimore corridor, a geographic location in suburban Maryland with limited mass transit, and a relative dearth of loco-regional industry jobs if workers later wish to transition out of government. Thus, despite the positive, mission-driven nature of FDA regulatory work, talent acquisition remains a significant challenge.

## History and recent performance of the Third Party Review program

**Fig. 2:**
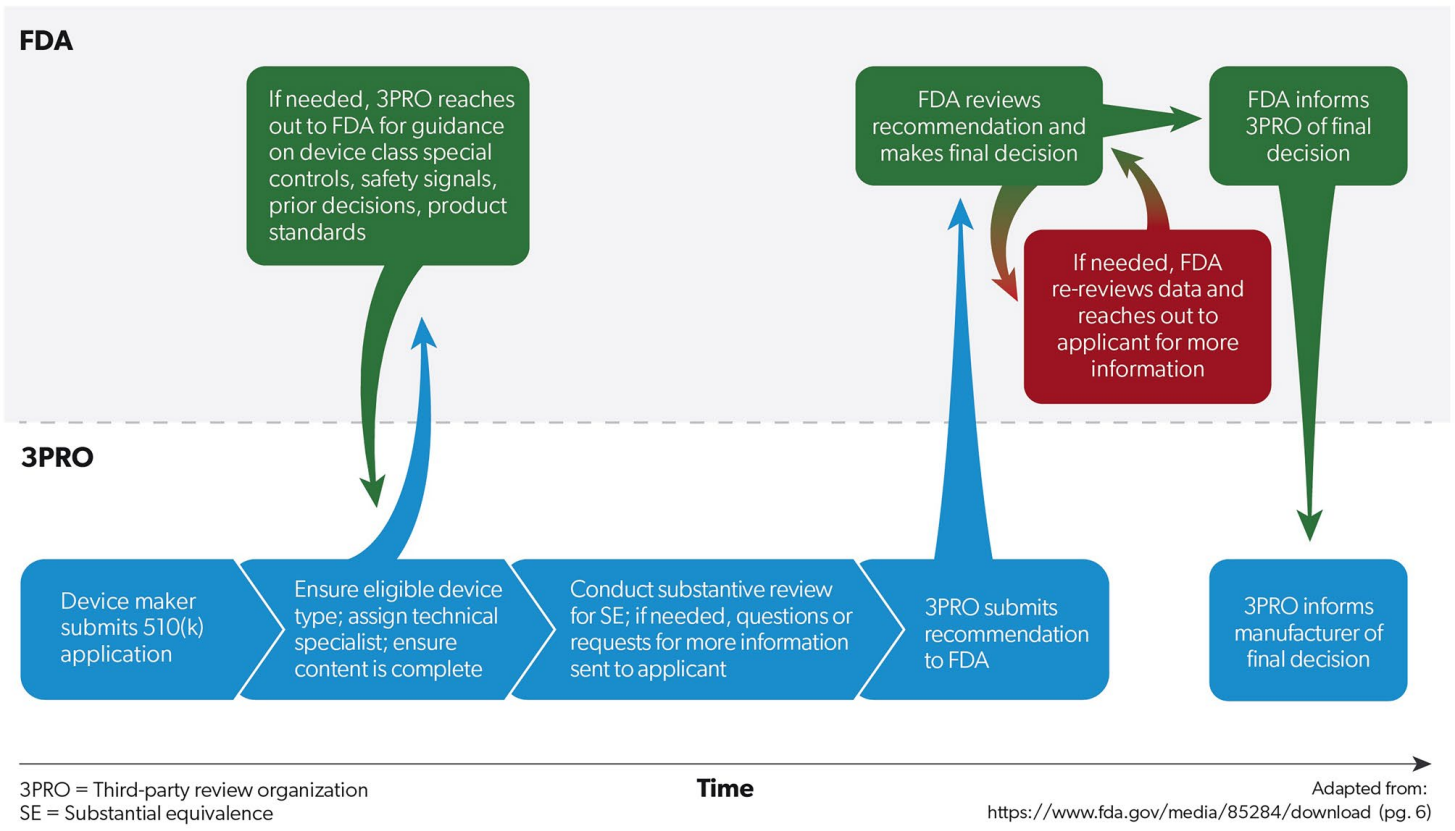
Third Party 510(k) workflow. When a device manufacturer decides to submit a 510(k) application to a third party review organization (3PRO), the organization first determines whether the device is within the proper class and type eligible for third party review. After ensuring the application has the appropriate content, it reaches out to the FDA prior to its substantive review if it needs more background information on the approval standards such as class-specific special controls. Following its substantive review, the 3PRO determines whether the application has met the requirements to find substantial equivalence (SE) to a predicate device and submits its recommendation to the FDA. If the FDA reviews the 3PRO’s decision and determines it needs more information, the FDA will reach out to the applicant directly. As the 3PRO’s are supposed to be the sole conduit of communication with the applicants, when the FDA has to reach out to the applicant, it classifies the application as under “re-review.” Once the FDA has made its final decision (with or without re-review) it informs the 3PRO

Recognizing the at times overwhelming volume of review, as part of the 1997 FDA Modernization Act (FDAMA), Congress created the 510(k) Third Party Review program (see Fig. 2). Functionally, the goal was to offload the review of low complexity devices with low- or moderate-risk to recognized third party review organizations in order to free up agency staff time to address more complex applications and cutting-edge regulatory issues by giving device manufacturers the option to submit its application to an accredited third party review organization. Class III devices (i.e., those with the highest risk profile), devices requiring a De Novo submission (i.e., those without an existing predicate), as well as devices intended to be permanently implantable, life-sustaining, or life supporting are all statutorily excluded from third party review and must be submitted to the FDA [34]. The program has been subject to continued legislative attention and revision, with the 2012 FDASIA (MDUFA III) adding a 3-year reaccreditation cycle for review organizations, and the 2017 FDARA (MDUFA IV) directing the FDA to issue guidance regarding the types of devices eligible for third party review. The agency has attempted to support the program, recognizing that investments in quality, training, and vendor management can promote expedient scaling of the capacity of third party review organizations in a more efficient fashion than scaling internal review of low and some moderate-risk products.

Subsequently, the agency’s March 2020 guidance [35] enumerated eligible device types and elaborated on factors that would make a device ineligible for third party review such as combination products, novel cross-labeling considerations, or post-market data suggesting that the device type is the subject of a recent safety communication, high-risk recall, or safety signal. The guidance also clearly delineated requirements for recognition of third party review organizations, in addition to basal process suggestions for operating a recognized review organization. The program occasionally struggled with re-review, wherein FDA reviewers had to reach out to the device manufacturer/applicant directly and thereby repeat the device product review that the review organization had just completed. Consequently, 2017 FDARA Act (MDUFA IV) [36] included a commitment to audit for agency re-review and re-training by creating and implementing a plan to eliminate routine agency re-review of third party reviews [37], something that was reiterated in the Food and Drug Omnibus Reform Act of 2022 (“FDORA”; MDUFA V) [38] and its corresponding commitment letter from the FDA [39].

Despite the best of intentions, the third party review program has struggled. Utilization declined from a peak of 9.3% in 2008 to 2.4% in 2020,^4^ a decline due to a multitude of factors. First, potentially low quality reviews by third party review organizations routinely lead to FDA re-reviews [40], an issue that became increasingly common during the pandemic (2021–2022) [41]. FDA decision-making on third-party reviews slowed while re-reviews increased, with the share of applications “pending final decision” increasing from 0% in FY2018-2020 to 8% in 2021 to 30% in 2022 [42]. While re-review was the target of elimination in the FDA’s 2018 plan [43], the recent 2020 program guidance simply refers to the 2018 plan without acknowledging increasing program performance challenges.

Industry concerns about the third party review program grew with the August 2021 withdrawal of the accreditation of “Accelerated Device Approval Services” (ADAS) due to the organization fraudulently representing the identification, qualifications, and signature of its final reviewer and for misleading its client about ADAS’ communications with the FDA [44]. Consequently, the market for recognized review organizations decreased from ten to nine organizations [45]. While the reasons for challenges with the third party review program are myriad, policy experts and public health advocates have expressed healthy concern regarding the program [46] due to the removal of an accredited organization for fraud, low programmatic utilization, and frequent FDA re-review of applications.

## Stakeholder analysis: Unleashing the potential of Third Party Review

**Fig. 3:**
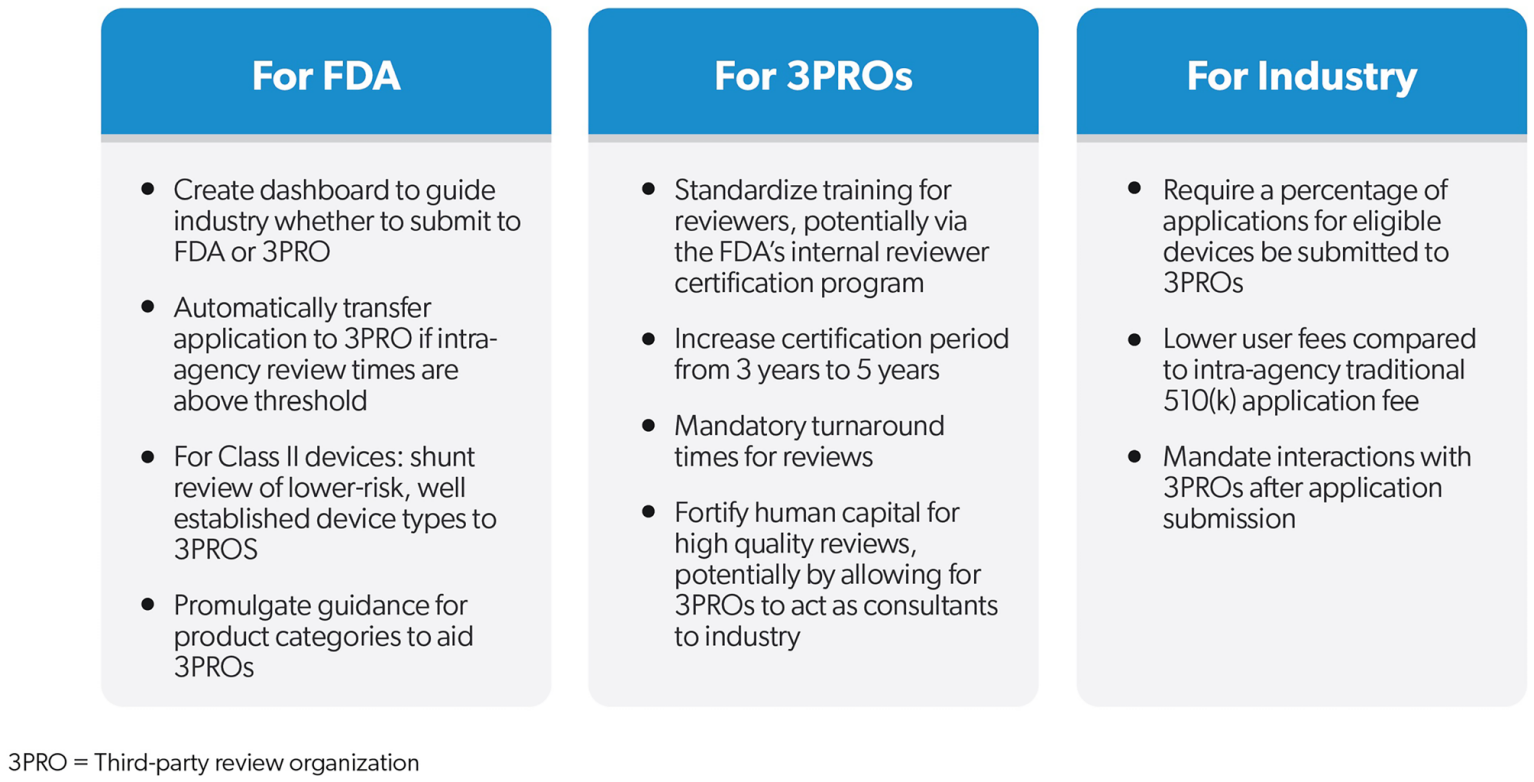
Policy proposals for the Third Party 510(k) program. To increasing the efficiency of third party reviews of 510(k) applications, the following policy proposals are directed at the major stakeholders: FDA, the third party review organizations (3PROs), and the medical device industry. Overall, these proposals aim to streamline the review of low-to-moderate risk device with less technological complexity by 3PROs. Improving the training and certification processes and retaining human capital within the 3PROs, as well as mandating that user fees and timelines for review are favorable to intra-FDA pathways (see Figure 1), 510(k) reviews for certain devices should be automatically shunted (or original applications mandated) through the 3PROs. Ultimately, this will allow for FDA to focus its regulatory attention to higher risk devices and those with newer, cutting edge technology, while still ensuring high quality review for the protection of patients.

With the 510(k) third party review the subject of scrutiny, policymakers have a window of opportunity to reform and revitalize the program, thus improving the overall 510(k) review program for all stakeholders (see Fig. 3). FDA could gain by decreasing the need for re-review, improving the quality of third party reviews, and by crafting a channel to decrease agency review volume for low and low-moderate complexity devices. In times of uncommonly high volume, such as during the COVID-19 pandemic when two reviewers committed suicide due to excessive review workload [47], the agency could have rapidly titrated up its review capacity. Medical device manufacturers would also gain from programmatic improvements, with small or mid-size manufacturers gaining an option for closer guidance on the review process and filing while scaled review of low and select moderate complexity devices could lower costs and increase review speed for large manufacturers. Finally, recognized review organizations could benefit from certainty of review volume, improving staff retention and quality of reviews, and improved staff training and information sharing of predicate reviews from FDA. We review policy improvements from the perspective of each stakeholder in turn.

The FDA completes review of ~3,000 510(k) applications annually, a number that has been largely stable since 2007. The agency, faced with an overwhelming volume of low and moderate complexity applications along with increasing intricacy and uncertainty in emerging regulatory areas such AI/ML, has multiple policy options. Firstly, the agency could mandate that a minimum percentage of eligible devices or entire specific devices classes or categories default to third party review instead of the FDA. Mandating specific device classes (e.g. class I) or product categories (e.g. facemasks) would drive volume and improve both reviewer quality and manufacturer experience with review organizations, albeit at the potential cost of removing the experience from FDA reviewers. Alternatively, directing a minimum percentage of a device product category to review organizations presents a more pragmatic alternative, also providing the agency with a built-in “flex up” option to handle surges in volume. This may require a “ceiling” market share (i.e. a minimum, pre-specified FDA review market share that should be maintained when third party review volume reaches a certain level) in order to ensure that FDA reviewers retain experience with the product category.

In order to provide an automatic pressure release valve for reviewers, when review volume reaches a certain level or the delay in the median or mean 510(k) review time for an established product category reaches a pre-determined threshold, subsequent applications would be automatically shunted to an appropriate review organization. The FDA could also consider specific targeted channeling, such as directing class I devices to an appropriate review organization or even screening all class II devices internally at CDRH with divided distribution between FDA internal review and to a set of qualified. specific third party review organizations. The third party review program can also be used to further stratify class II devices, addressing the concerns of critics who note challenges with the breadth of the class II designation [23]. In doing so, the agency could remove eligibility for third-party review for some class II devices with potential patient safety concerns (e.g. continuous ventilator [48–50], electrosurgical equipment [51–53]) and those subject to stronger post-market surveillance measures. That is, third party review of class II devices should be driven primarily by standards and less so by reviewer judgment. To facilitate third party review, the FDA could consider developing more device product category-specific guidance documents in addition to specifying circumstances that would result in the transfer of a product category back to in-agency review, such as recall rates or the severity of reported adverse events, noting that adverse event rates could be used to further scope class II devices (i.e. a living system of exclusionary criteria rather than inclusionary criteria for third party review eligibility).

Recognized review organizations face distinct challenges. Human capital management – recruiting and retention – remains a challenge, with the aforementioned structural proposals providing a guaranteed review volume promoting talent recruitment and retention. Reviewer training remains a challenge, with review organizations having access to a limited set of reviews through CDRH learn [54], a public web site with information about the medical device review process for industry. Instead, with the appropriate addition of privacy and data security protections, the FDA could consider providing access to the FDA’s Internal Reviewer Certification Program for third party reviewers, reducing agency effort by eliminating the need to maintain dual training programs with differing content. While likely requiring statutory change, the agency could publish – behind a firewall – a specified minimum number of redacted review memos for each product category, facilitating reviewer learning. Alternatively, the agency could give reviewers from third party organization access to all reviews but only for their predicate device.

Policymakers could require the FDA to market the program and update the agency web site with a clear dashboard inclusive of third party review organization performance metrics, allowing review organizations to compete on the basis of speed, clearance rates, and other metrics. Akin to travel web sites like booking.com or the Medicare plan-finder web site where consumers can enter in specific prescription drugs to determine their coverage options, the FDA should maintain a directory of review organizations with the ability for manufacturers and device entrepreneurs to search by product category, reducing manufacturer search burdens and review organization administrative effort answering queries regarding products that they do not cover. To facilitate stability of organizational recognition and reduce administrative burdens, the FDA should change the recognition cycle for review organizations from three to five years, with the initial recognition period lasting seven years inclusive of a mandatory two year “shoulder-to-shoulder” period of close interaction with CDRH for training purposes. FDA should also consider recognizing review organizations for a specific product category or categories, helping ensure honest marketing by review organizations and providing assurance to manufacturers undergoing third party review. Policymakers can also support the FDA in fighting bias in reviews noting that once accepted, 510(k) application fees are nonrefundable [55]. A similar policy could be implemented for third party review organizations as a mechanism to remove incentives for lowering market entry standards.

Finally, industry or medical device manufacturers face multiple challenges in utilizing the third party review program noting that it is currently focused on process and not efficient and effective review. Like the pharmaceutical industry [56], each day under review incurs economic opportunity cost for device manufacturers. Policymakers could consider lowering regulatory costs for manufacturers through a variety of levers, including making third party review timelines explicit, binding to the agency, by decreasing the FDA decision time from 30 to 21 days, making the FDA review timeline binding through a statutory requirement or commitment letter, or making third party reviews binding for certain select device products except in case of review process defects. Other alternatives include FDA implementation a soft cap for third party review at 60 days for class I non-exempt devices.

Policymakers could directly lower regulatory costs by capping review fees to some fraction of what would have been paid to the agency in user fees for the equivalent application review (e.g. 80%) if the volume of reviews exceeds a significant fraction (e.g. 50%) of the total volume in a product category. However, over-specification in statute may prevent review organizations from offering “concierge-level” service to new or small device manufacturers and prevent experienced manufacturers from choosing the desired level of service. Given their unique focused structure, review organizations can have more detailed and frequent interactions with manufacturers, functioning as a guide and adviser through the review process. Policymakers could also attach a regulatory incentive, akin to the priority review voucher program that exists for specific enumerated types of pharmaceutical products targeting medical countermeasures, tropical diseases, or rare pediatric diseases. A current barrier to small manufacturer program participation is a prohibition on third party review organizations from undertaking regulatory consulting [57], a policy put in place in order to prevent cross-pollination from the review function. Internal corporate firewalls to separate review and consulting may allow review organizations to better assist small companies, which the FDA typically defines as <$30 M in annual revenue [58]. Providing third party review organizations the opportunity to have multiple streams of work could also assist with attracting and retaining technically-trained and skilled human capital.

## Concluding Thoughts

The 510(k) program represents the majority of device review activities at the FDA, with multiple challenges including review volume, timeliness, and predicate device creep. Policymakers created, with assistance from the FDA and industry, the third party review program to offload agency burdens while increasing the speed of and reducing the costs of review. Unfortunately, the program has floundered for a variety of reasons, with agency re-review revealing challenges and a recent case of review organization fraud bringing important questions to the forefront of policy debate. Revitalizing the third party review program offers an opportunity to improve incremental device innovation in well-worn and low-to-moderate risk medical device product categories, freeing up the FDA to focus on new regulatory areas such as artificial intelligence/machine learning.

Thoughtful, stepwise, and incremental reform over time of the third party review pathway would empower the FDA to identify, address, and manage predicate device creep, and transform third party review organizations into a “peer review” operator for small companies due to their more nimble nature. While many programmatic improvements and policy changes will require legislative intervention, administrative agency support, and intervention over time, the benefits are significant. Over the past century, pharmaceutical product innovation has changed the course of human disease. Device and technology will shape the next century of medical care and policymakers must prepare the FDA for this future.

## Data Availability

No data were used in this study as it is a review paper.
